# Checkpoint effects and telomere amplification during DNA re-replication in fission yeast

**DOI:** 10.1186/1471-2199-8-119

**Published:** 2007-12-21

**Authors:** Katie L Mickle, Anna Oliva, Joel A Huberman, Janet Leatherwood

**Affiliations:** 1 Department of Microbiology and Molecular Genetics, SUNY at Stony Brook, Stony Brook, New York 11794-5222, USA; 2 Department of Cancer Biology, Roswell Park Cancer Institute, Buffalo, New York 14263-0001, USA

## Abstract

**Background:**

Although much is known about molecular mechanisms that prevent re-initiation of DNA replication on newly replicated DNA during a single cell cycle, knowledge is sparse regarding the regions that are most susceptible to re-replication when those mechanisms are bypassed and regarding the extents to which checkpoint pathways modulate re-replication. We used microarrays to learn more about these issues in wild-type and checkpoint-mutant cells of the fission yeast, *Schizosaccharomyces pombe*.

**Results:**

We found that over-expressing a non-phosphorylatable form of the replication-initiation protein, Cdc18 (known as Cdc6 in other eukaryotes), drove re-replication of DNA sequences genome-wide, rather than forcing high level amplification of just a few sequences. Moderate variations in extents of re-replication generated regions spanning hundreds of kilobases that were amplified (or not) ~2-fold more (or less) than average. However, these regions showed little correlation with replication origins used during S phase. The extents and locations of amplified regions in cells deleted for the checkpoint genes encoding Rad3 (ortholog of human ATR and budding yeast Mec1) and Cds1 (ortholog of human Chk2 and budding yeast Rad53) were similar to those in wild-type cells. Relatively minor but distinct effects, including increased re-replication of heterochromatic regions, were found specifically in cells lacking Rad3. These might be due to Cds1-independent roles for Rad3 in regulating re-replication and/or due to the fact that cells lacking Rad3 continued to divide during re-replication, unlike wild-type cells or cells lacking Cds1. In both wild-type and checkpoint-mutant cells, regions near telomeres were particularly susceptible to re-replication. Highly re-replicated telomere-proximal regions (50–100 kb) were, in each case, followed by some of the least re-replicated DNA in the genome.

**Conclusion:**

The origins used, and the extent of replication fork progression, during re-replication are largely independent of the replication and DNA-damage checkpoint pathways mediated by Cds1 and Rad3. The fission yeast pattern of telomere-proximal amplification adjacent to a region of under-replication has also been seen in the distantly-related budding yeast, which suggests that subtelomeric sequences may be a promising place to look for DNA re-replication in other organisms.

## Background

Two key functions of the cell cycle machinery are to ensure (i) that DNA replication is completed before cells enter mitosis and (ii) that DNA replication is limited to once per cell cycle such that re-initiation of DNA replication does not occur on newly replicated DNA until after cells have passed through mitosis. Thus DNA replication is tightly controlled to ensure that the genome is copied once and only once within a cell cycle.

DNA replication is regulated at three successive steps: (i) binding of proteins necessary for initiation onto DNA during the M and G1 phases of the cell cycle, (ii) initiation at origins during S phase, and (iii) imposition of re-replication restraints during the S and G2 phases.

In the fission yeast, *Schizosaccharomyces pombe*, overall control of replication is provided by the cyclin-dependent kinase, Cdc2 (Cdk1), whose activity drives the cell cycle. Low Cdk1 activity during late M phase and G1 phase permits the first step of replication, formation of pre-replication complexes (pre-RCs) at replication origins. Pre-RCs include ORC proteins (Orc1–Orc6) together with Cdc18, Cdt1, and MCM proteins (Mcm2–Mcm7). At the end of G1, Cdk1 activity begins to increase, triggering the second step: initiation of replication at subsets of pre-RCs throughout S phase (reviewed in [1]).

Once S phase has begun, the relatively high level of Cdk1 activity inhibits formation of new pre-RCs and thus inhibits re-initiation on already-replicated segments of DNA. As S phase continues, Cdk1 activity increases further, strengthening the inhibition of re-replication. In fission yeast, active Cdk1 inhibits new pre-RC formation by multiple pathways, including direct phosphorylation of Cdc18 [2,3] and Orc2 [4,5] and destruction of Cdc18 [6] and Cdt1 [7,8]. Similarly, in budding yeast multiple Cdk1-dependent pathways inhibit re-replication by preventing the formation of new pre-RCs. These pathways include Cdk1-dependent phosphorylation of Orc2, Orc6, and Cdc6 (Cdc18 homologue), destruction of Cdc6, and nuclear export of Cdt1 and Mcm2-7 (reviewed in [9]). Related pathways similarly limit DNA replication to once per cell cycle in metazoans (reviewed in [9]). Even though cell cycle progression and Cdk1 activate multiple pathways that restrict DNA replication to once per cell cycle, these controls can be disrupted in *S. pombe *by high level expression of a single protein, Cdc18 [2,10,6].

Two-dimensional (2-D) gel analysis has shown that some re-replication events begin at sequences previously identified as S-phase origins in fission yeast [11], and both 2-D gel analyses [12,13] and genome-wide microarray studies of copy number changes [12,14] have shown that, as expected, re-replication also begins at sequences previously identified as S-phase origins in budding yeast. Even though re-replication appears to be initiated from sites that also function as S-phase replication origins, two genome-wide studies in budding yeast have both concluded that origin use during re-replication is distinctly different from origin timing or efficiency during normal S phase [12,14]. Regions that are re-replicated in fission yeast have yet to be determined, as the only origin so far tested during re-replication in fission yeast is that of ribosomal DNA, *ars3001 *[11].

In budding yeast, when Cdk1 phosphorylation sites in Orc2, Orc6, Cdc6, and MCMs are mutated or circumvented and Cdc6 is over-expressed, DNA re-replication is limited to a maximum DNA accumulation of ~3–4C in haploid cells [12-15]. Even after all known mechanisms to prevent re-replication in budding yeast have been eliminated, multiple restraints remain. First, pre-RCs re-assemble at only a subset of the sites used for a normal S phase [14]. Second, of these only a subset are selected for replication re-initiation [12,14]. Finally, there is some evidence that fork processivity may be reduced during re-replication [12]. The mechanisms for these remaining restraints are unknown. They may be responsible for the budding yeast differences between S-phase replication and re-replication. It has also been suggested by Tanny *et al*. that differences in chromatin organization and/or gene expression between S phase and G2 may change origin selection and/or efficiency between S-phase replication and re-replication [12,14].

In *S. pombe*, over-expression of Cdc18 is sufficient to drive re-replication and this re-replication can be further increased by mutating phosphorylation sites in Cdc18, by also mutating phosphorylation sites in Orc2, or by simultaneously over-expressing Cdt1 [7,3,4,11]. Reasons for the different sensitivities of fission yeast and budding yeast to re-replication are unclear.

In budding yeast, re-replication leads to double-stranded DNA (dsDNA) breaks and activation of checkpoint protein Rad53 that is dependent on DNA damage response proteins [16-18]. In *X. laevis *egg extracts, re-replication induced by addition of recombinant Cdt1 leads to activation of Chk1 (the effector kinase used during DNA damage and replication blocks) and the presence of small dsDNA fragments [19,20]. In human cells, re-replication induced by (i) inactivation or depletion of the Cdt1 inhibitor, Geminin [21-23], (ii) depletion of proteins integral for the degradation of Cdt1, DDB1 or Cdt2 [24,25], (iii) depletion of Emi1 (an inhibitor of APC/C activity during S and G2 phases) [26], or (iv) over-expression of Cdt1 and Cdc6 with cyclinA-Cdk2 [27] activates the ATM/ATR/Chk2 DNA damage pathways. Accumulation of single-stranded and double-stranded DNA has also been observed during re-replication [21].

It is possible that this re-replication-induced checkpoint activation in turn influences the patterns of re-replication. The relation between checkpoints and origin selection during re-replication is unknown. DNA-replication and DNA-damage checkpoint pathways are highly conserved among eukaryotes. The fission yeast Rad3 checkpoint kinase (product of the *rad3 *gene) is a homologue of vertebrate ATR and of budding yeast Mec1. Rad3 is activated in response to blocked replication forks or DNA damage. The Cds1 checkpoint kinase (product of the *cds1 *gene) is activated after phosphorylation by Rad3 in response to stalled replication forks or DNA damage during S phase. Cds1 is a homologue of vertebrate Chk2/Cds1 and of budding yeast Rad53. A different kinase, Chk1 (product of the *chk1 *gene) is activated by Rad3 in response to DNA damage in late S and G2 phases. Chk1 homologues, with the same name, are also present in vertebrates and in budding yeast (reviewed in [28]).

In this study, we drove re-replication in *S. pombe *by over-expression of a mutant Cdc18 protein and then used microarrays to find out if specific regions in the genome are preferentially amplified during re-replication. We then repeated these experiments in *cds1*Δ and *rad3*Δ cells to find out if checkpoint responses determine regional susceptibility to re-replication.

## Results

### Over-expression of cdc18* increases DNA content ~four-fold

**Figure 1:**
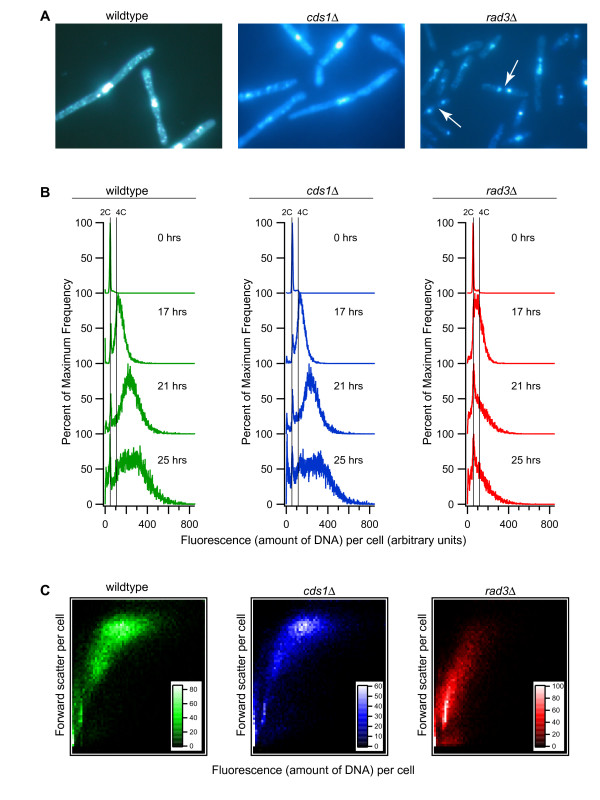
Effect of inducing cdc18* on DNA content and cell cycle division arrest. cdc18* was induced in the wild-type (green), *cds1*Δ (blue), and *rad3*Δ (red) strains by removal of thiamine at 0 hours. (A) Fluorescence microscopy of cells 25 hours after cdc18* induction. Cells are stained with DAPI to show nuclei. Arrows show binucleate *rad3*Δ cells, which are evidence for active cell division. (B) Histogram plots of DNA content determined by flow cytometry for 0, 17, 21, and 25 hours after removal of thiamine. (C) Density plots of DNA content (fluorescence intensity, x-axis) versus cell size (forward scatter, y-axis) at 25 hours. Wild-type and *cds1*Δ cells are greatly elongated with high DNA content whereas *rad3*Δ cells are shorter and have lower DNA content due to cell division during re-replication. The insets show the number of cells out of 10,000 represented by pixels of the indicated color.

Re-replication in our experiments was driven by inducing the cdc18* gene, encoding the Cdc18* protein, from an integrated allele regulated by the *nmt1 *promoter. Cdc18* lacks Cdk1 phosphorylation sites at positions 26, 98, 104 and 134 [3]. Phosphorylation of these sites is important for Cdk1 downregulation of Cdc18 function, for targeting Cdc18 for proteolysis [2-4] and probably also for Cdc18 inhibition of Cdk1 ([29] and A. Vas and J. L., unpublished results). A major goal of this study was to compare re-replication in wild-type cells with re-replication in checkpoint mutant cells. Figure 1 describes the effects of Cdc18* overexpression on cell morphology, cell cycle arrest, and DNA re-replication in wild-type as well as *cds1*Δ and *rad3*Δ mutants. In the strains studied here, *cdc18+ *remains at its normal locus, and cells grow normally as long as cdc18* is repressed. Removing thiamine (B1) from the medium turned on the *nmt1 *promoter and induced cdc18*. Cdc18* protein was detectable by 13 hours (not shown), and re-replication was detectable by 17 hours ([4] and Figure 1).

Cdc18*-driven re-replication doubled the DNA content per cell from 2C (haploid cells after S-phase) to an average value > 4C (in wild-type and *cds1*Δ cells) or ~4C (in *rad3*Δ cells) by 17 hours as determined by flow cytometry (Figure 1B). Though highly variable from cell to cell, the DNA content of wild-type and *cds1*Δ cells approximately doubled again between 17 and 21 hours. The average DNA content of *rad3*Δ cells increased only slightly if at all during this time interval. From 21 to 25 hours, there was increased variability but little change in average DNA content per cell in all three strains. The flow cytometry profiles are consistent with DNA re-replication models ranging from DNA accumulation by repeated re-replication of a few regions of the genome to continuous, albeit slow, whole genome re-replication. If the re-replicating cells are in a continuous "re-replication" phase, then DNA re-replication is inefficient compared with normal S-phase replication, which doubles DNA content in 20–40 minutes under growth conditions similar to those used here.

### Use of microarrays to determine which regions are re-replicated

We used microarray analyses of DNA copy number – a procedure which has come to be called comparative genomic hybridization (CGH) – to distinguish between the possibility that re-replication affects sequences genome-wide and the possibility that re-replication is limited to a small subset of sequences and drives DNA accumulation by high amplification, onion-skin-like replication bubbles similar to those generated during chorion gene amplification in *Drosophila melanogaster *(reviewed in [30]). If re-replication only affected a few regions in the genome – for instance, if only half of genomic sequences were susceptible to re-replication – then, by 21 hours when the total amount of DNA is increased four-fold, we would expect an eight-fold difference in copy number between re-replicated DNA regions and the regions that are not re-replicated. On the other hand, if re-replication occurred uniformly genome-wide, we would expect little or no variation in copy number from region to region no matter how long re-replication continued.

**Figure 2:**
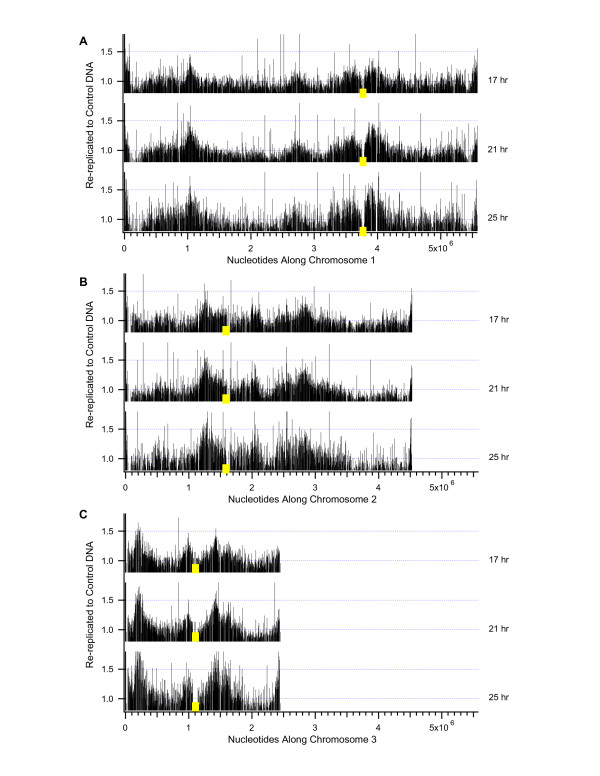
**Genome-wide microarray analysis of DNA re-replication over time in wild-type cells**. The relative amplification of sequences was determined by competitive hybridization of DNA from re-replicating cells against DNA from the same strain prior to re-replication. DNA re-replication profiles of wild-type cells induced for 17 hours, 21 hours, and 25 hours are shown for chromosomes 1 (A), 2 (B), and 3 (C). Centromeres are indicated by yellow squares. No smoothing of data has been applied. Vertical lines indicate the relative level of DNA amplification across the genome. A value of 1.0 is the average amount of re-replicated DNA for the genome. Values greater than 1.0 represent probes which were replicated more than the average amount of re-replication. The results reveal a gradual increase in differences between the most amplified and least amplified regions over time.

To determine relative copy number, DNAs harvested from re-replicating cells at 17, 21, and 25 hours after cdc18* induction (re-replicated) and DNA from the same strain at 0 hours, when cdc18* was repressed (control), were labeled and hybridized competitively to microarrays. Overall ratios of re-replicated DNA to control DNA were normalized to 1.0. Thus if a probe sequence was re-replicated an average amount, its ratio would be 1.0 even if the genome was four-fold over-replicated (~8C). Relatively amplified sequences would have ratios greater than 1.0, and regions that failed to re-replicate or re-replicated less than average would have ratios less than 1.0. Individual probe values from replicate hybridizations were averaged and graphed for the 17-, 21-, and 25-hour time points, without smoothing or averaging of neighboring probe values (Figure 2). Probe values are provided in Additional Files 1 (chromosome 1), 2 (chromosome 2), and 3 (chromosome 3).

DNA re-replication varied regionally such that stretches of hundreds of kilobases were relatively amplified during re-replication or were relatively under-re-replicated. Wild-type results are shown in Figure 2. Results for the *cds1*Δ and *rad3*Δ strains are in Additional Files 4 and 5. Some of the probes appeared to re-replicate significantly more or less than the other probes in their regions, giving rise to the sharp spikes and valleys evident in Figure 2. We are not yet certain of the significance of these spikes and valleys. They may represent noise, or they may represent "hot spots" of over- or under-replication. They are the subject of continuing investigation. For this reason, in the remainder of this manuscript we shall focus on the regional variations and ignore the spikes and valleys.

Regional amplification increased somewhat between 17 and 21 hours and changed little between 21 and 25 hours. The degree of relative amplification was generally modest (~1.5 fold), and the regions of amplification remained stable over time. The highest level of amplification represented by multiple adjacent probes was ~2-fold. Therefore no individual regions consistently re-replicated to very high ploidies, and most of the genome was re-replicated somewhat. A few regions represented by multiple probes were under-represented by ~1.7-fold and therefore failed to re-replicate in many cells. Microarray analysis of amplification during re-replication was highly reproducible for all three time points.

Since some regions were re-replicated more than two-fold over others, the re-replicating cells must contain replication forks and/or double-strand breaks. It is expected that such structures will activate DNA-replication and/or DNA-damage checkpoint pathways. Given the importance of DNA-replication and DNA-damage checkpoint responses for genome stability and the roles of these pathways in controlling replication initiation and fork processivity, we next sought to determine to what extent these pathways regulate, modify, or restrain DNA re-replication in *S. pombe*.

### Roles of Rad3 and Cds1 in cell cycle arrest during DNA re-replication

To find out how replication-checkpoint and DNA-damage-response signals affect DNA re-replication in *S. pombe*, inducible cdc18* was combined with deletions of the *cds1 *and *rad3 *genes. Like the cdc18* strain (hereafter referred to as "wild-type"), the cdc18* cds1Δ and cdc18* rad3Δ double-mutant strains (hereafter referred to as "*cds1*Δ" and "*rad3*Δ") are viable when cdc18* is repressed, and all three strains show 90–95% loss in viability 17 hours after cdc18* induction (data not shown). Deletion of *cds1 *abrogates the replication checkpoint, and Cds1 is important for maintaining functional replication forks during replication stress (for example when dNTPs are limited, as happens when cells are treated with HU). Cds1 is able to arrest the cell cycle in HU-treated cells, but Cds1 is not required for this cell cycle arrest, because the DNA-damage responsive Chk1 kinase has overlapping functions with Cds1 and is also able to arrest the cell cycle. The Rad3 kinase (related to ATR and Mec1) is required for both the Cds1 and Chk1 pathways. Thus cells lacking Rad3 cannot arrest the cell cycle in response to either replication stress or DNA damage.

Over-expression of Cdc18* was sufficient for initial cell-cycle arrest in all three strains studied here, including the *rad3*Δ strain; however, maintenance of the Cdc18* cell-cycle arrest was strongly dependent on Rad3 and partially dependent on Cds1. Fission yeast in a cell-cycle arrest become highly elongated, remain uni-nucleate, and lack septa. At 17 hours, the wild-type cells remained fully cell-cycle arrested. Fewer than 1% of these cells had septa or were bi-nucleate (based on fluorescence microscopy of DAPI- and Calcafluor-stained cells; Figure 1A), and >90% of these cells were highly elongated. At 17 hours, most of the *cds1*Δ cells also maintained the cell cycle arrest (2% bi-nucleate). In contrast, by 17 hours, the *rad3*Δ cells had resumed division (15% bi-nucleate cells), and due to the failure to maintain cell cycle arrest, only 53% of the *rad3*Δ cells were highly elongated, unseptated, and uni-nucleate. By 21 hours, 5% of wild-type, 11% of *cds1*Δ, and 16% of *rad3*Δ cells were bi-nucleate. Thus, by 21 hours even wild-type cells were imperfect in maintaining the cell cycle arrest, and the *cds1*Δ strain had a clear arrest defect in a small subset of cells (Additional File 6).

**Figure 3:**
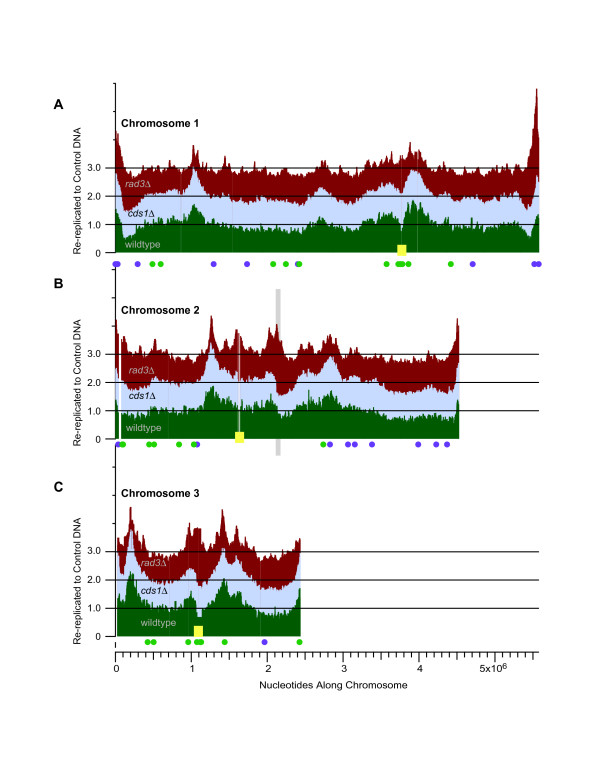
**Comparison of re-replication between the wild-type and checkpoint-mutant strains**. DNA re-replication profiles from the wild-type (dark green), *cds1*Δ (light blue), and *rad3*Δ (dark red) strains induced for Cdc18* over-expression are shown for chromosomes 1 (A), 2 (B), and 3 (C). Median probe values across 5 neighboring probes from the 21- and 25-hour data sets were used to smooth data into one composite DNA re-replication profile for each strain along each chromosome. In order to view all three re-replication profiles for a chromosome, the *cds1*Δ data are offset on the Y-axis by +1.0 and the *rad3*Δ data are offset by +2.0. Centromeres are indicated by yellow squares. The mating-type locus on chromosome 2 is marked by a light grey vertical rectangle. Plotted as solid circles below each chromosome are origins which fired with greater efficiency in the *cds1*Δ and *rad3*Δ (purple) or wild-type (green) strains when cells were treated with HU in the previous study by Mickle *et al*. [31]. Notice that all of the large amplified regions found in wild-type cells are also found in *cds1*Δ and *rad3*Δ cells. *rad3*Δ cells have additional small amplified regions which are not present in the wild-type or *cds1*Δ strains. Thus checkpoint proteins do not affect origin selection during re-replication as they do during replication.

Our flow cytometric results also suggested that cell-cycle arrest requires Rad3. Figure 1C shows forward scatter (which reflects cell length) versus florescence per cell (which is proportional to DNA content). Most wild-type and *cds1*Δ cells were elongated and contained large amounts of DNA (~6–10C). However, *rad3*Δ cells were much shorter and had less DNA per cell than wild-type, as expected for cells that fail to maintain cell-cycle arrest (Figure 1C). Because a large fraction of *rad3*Δ cells were dividing, flow cytometry cannot be used to quantitate the extent of re-replication driven by Cdc18* in this strain. However, we are confident that re-replication took place, because (i) DNA content was greater than 2C for the majority of cells at 17–25 hours (Figure 1B), (ii) the pattern of over-replication measured by microarray analysis was similar to that of wild-type and *cds1*Δ cells (Figure 3), and (iii) cells lost viability similarly to wild-type. Based on microscopy of DAPI-stained cells, most divisions in *rad3*Δ cells at 17 hours and later were aberrant, with unequal divisions of nuclei as well as strings of DNA between divided nuclei (Figure 1A). Such aberrant divisions could be generated by attempting to segregate chromosomes with partially re-replicated DNA.

### Regional amplification is largely independent of Rad3 and Cds1

Genomic DNAs from re-replicating *cds1*Δ and *rad3*Δ cells were labeled and hybridized against genomic DNA from wild-type cellswith cdc18* repressed (control). Hybridization results were normalized to 1.0 for each array, triplicate hybridizations were averaged, and individual probe values were graphed along each chromosome for 17, 21, and 25 hours (*cds1*Δ, Additional File 4; *rad3*Δ, Additional File 5) and are provided in Additional Files 1 (chromosome 1), 2 (chromosome 2), and 3 (chromosome 3). As for wild-type cells, *cds1*Δ and *rad3*Δ profiles were highly reproducible at the 3 time points, with the magnitude of the effects slightly greater at 21 and 25 hours than at 17 hours.

The amplified regions were similar in all three strains at all three times as seen by comparing the nine plots for each chromosome shown in Figure 2 and Additional Files 4 and 5. To show comparisons more simply, the highly similar 21 and 25 hour times were averaged and then plotted for each of the three strains in Figure 3. The wild-type and *cds1*Δ results are nearly identical. Distinct differences can be seen between the wild-type or *cds1*Δ strains and the *rad3*Δ strain, but the overall locations of amplified regions are generally the same. Exceptions are discussed below.

The similarity between the *cds1*Δ and wild-type strains (Figure 3) indicates that control of replication origin function by Cds1 has little effect on origin activity during re-replication. This was initially surprising to us. However, during the course of these studies, we found that Cds1 and Rad3 significantly restrain only about 3% of origins and significantly stimulate only about 5% of origins during S-phase replication in HU-treated fission yeast cells [31]. But even within that small subset of origins (3–5%), none was similarly affected by checkpoint mutations during re-replication (Figure 3). The striking similarity between re-replication in the *cds1*Δ and wild-type strains also suggests that replication fork movement is similar in these strains while they are undergoing re-replication. In contrast, when these strains are treated with HU during normal S phase, replication fork movement is much slower in *cds1*Δ (and in *rad3*Δ) cells than in wild-type cells [32].

We detected three distinct, but minor, differences between the patterns of re-replication in *cds1*Δ or wild-type cells and those in *rad3*Δ cells. First, if one carefully examines Figure 3, many small peaks of amplification are visible in the *rad3*Δ strain that are undetectable in the other two strains. These *rad3*Δ-specific peaks are correlated with locations of relatively strong origins in HU-treated cells. Second, the amplified regions in *rad3*Δ cells have more distinct boundaries than do the peaks in wild-type or *cds1*Δ cells. This might result from reduced fork rate or lower fork processivity in the *rad3*Δ strain.

Third, the telomeres of chromosomes 1 and 2, centromeres on all chromosomes, and the mating-type region on chromosome 2 are preferentially amplified in *rad3*Δ cells (Figure 3). In this respect these three types of heterochromatin behave similarly to each other – in contrast to normal S phase, where telomeric heterochromatin is late-replicating and checkpoint-regulated, while centromeric and mating-type heterochromatin are early-replicating and not affected by the replication checkpoint ([33,31]). Amplification of telomere regions is analyzed in more detail in the next section.

These results for the *rad3*Δ strain are preliminary because *rad3*Δ cells continued mitotic divisions during re-replication whereas wild-type and *cds1*Δ cells did not. Therefore, we cannot distinguish whether *rad3*Δ-specific effects are due to Rad3-specific functions or to interruption of re-replication by mitosis, possibly followed by an S phase.

### Amplification of telomeric regions during re-replication

**Figure 4:**
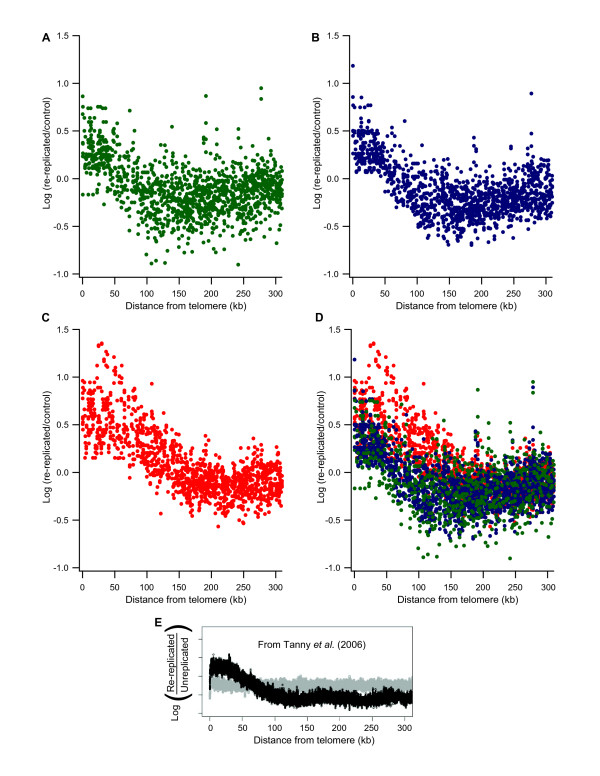
**Re-replication of regions adjacent to telomeres**. Probe values were plotted as a function of their distance from the closest telomere for chromosomes 1 and 2 in fission yeast or for all chromosomes in budding yeast (E). Chromosome 3, which has atypical telomeres due to the presence of rDNA repeats, was omitted from the analysis. Probe values for wild-type (A; green), *cds1*Δ (B; blue), *rad3*Δ (C; red), and all three strains (D; green, blue, and red) induced for Cdc18* over-expression at 17, 21, and 25 hours of Cdc18* induction are shown. Re-replication is enhanced up to 50 kb from the ends of telomeres in wild-type and *cds1*Δ cells and up to 100 kb in *rad3*Δ cells. (E)Re-replication of sub-telomeric regions up to 50 kb from the ends of chromosomes was also enhanced in checkpoint-competent *S. cerevisiae *cells as shown in this figure from Tanny *et al*. The figure shows relative enrichment for each spot on their microarray plotted as a function of its distance to the closest telomere for both the re-replicating strain (black) and wild-type strain (gray) [14].

Microarray copy number analyses indicate that *S. pombe *telomeric regions are amplified during re-replication. Our data show that replication forks must be initiated in telomeres or within the subtelomeric regions during re-replication. Telomere-associated amplification is evident in the wild-type and *cds1*Δ strains, and the effect is magnified in the *rad3*Δ strain, as described above and in Figure 3. To focus analysis on telomeric regions as a class, Figure 4 displays probe values as a function of distance from telomeres. Note that chromosome 3 is excluded from this analysis, because it terminates in rDNA repeats rather than in the telomere-associated sequences located at the ends of chromosomes 1 and 2. Also note that our arrays detect sequences near to the telomeres rather than telomere repeats themselves.

The wild-type and *cds1*Δ sub-telomeric amplified regions span 50–75 kb from the ends of chromosomes and are followed by under-re-replicated regions which extend out to ~300 kb from the chromosome ends (Figure 4A, B). The *rad3*Δ sub-telomeric amplified regions are larger, spanning up to 100–150 kb from the chromosome ends (Figure 4C,D). As in the wild-type and *cds1*Δ strains, the sub-telomeric amplified regions in *rad3*Δ cells are followed by under-re-replicated regions.

The pattern of re-replication near telomeres in *S. pombe *appears to be the same as re-replication near telomeres in the distantly related budding yeast, *S. cerevisiae*, when re-replication is induced in G2/M phase (Figure 4E). Analysis of re-replication in budding yeast showed re-replication of telomeric regions in which the terminal ~50 kb is amplified greater than average for the genome and the following >300 kb is under-re-replicated [14]. That this distinct pattern of re-replication adjacent to telomeres is conserved between the highly diverged fission and budding yeasts suggests that this may be a basic feature of re-replication near telomeres and therefore may also occur in metazoans.

### Origin use during re-replication is distinct from S-phase replication

**Figure 5:**
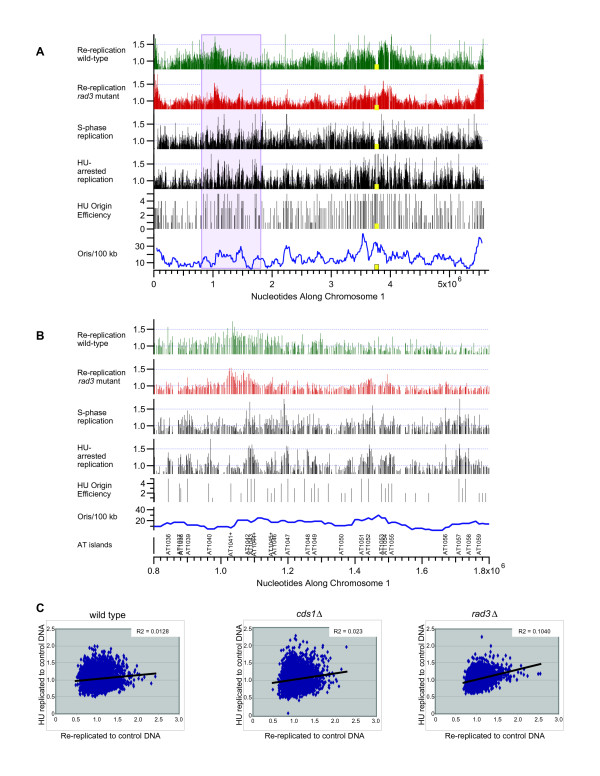
**Comparison of regions amplified during DNA re-replication with active S phase origins**. (A) From top to bottom, the panels show (for chromosome 1) the DNA re-replication profiles at 25 hours after thiamine removal of wild-type cells (green) and *rad3*Δ cells (red); the replication profile of wild-type cells replicating under normal conditions in the absence of HU; and the replication profile of wild-type cells replicating in the presence of HU. No smoothing of data was performed. The overall efficiencies of origin firing during HU treatment, from the studies by Mickle *et al*. [31], are plotted as black sticks below the HU-arrested replication profile. The lengths of the sticks represent the levels of efficiency of the origins during the HU treatment. The longer the stick, the more efficiently the origin fired. Note that the measure of overall efficiency employed by Mickle *et al*. combined efficiency in wild-type cells with efficiencies in checkpoint-mutant cells [31]. For this reason, telomeric origins in chromosomes 1 and 2 show relatively high efficiencies, even though the extents of replication at telomeres were small in wild-type cells. The bottom panel shows the cumulative sum of origin scores in a sliding 100-kb window. Centromeres are indicated by yellow squares. The region spanning 0.8 kb to 1.8 kb along chromosome 1 is highlighted by a light purple box. (B) A closer look at the highlighted amplified region (light purple box in (A)) shows that the pattern of re-replication is clearly different from patterns of replication. (C) The amount of replication under HU stress was compared to the amount of re-replication by plotting relative copy numbers under HU stress for 4 hours [31] against re-replicated to control DNA ratios for strains 25 hours after thiamine removal. Re-replicated to control DNA ratios and relative copy numbers for all probes were plotted for wild-type, *cds1*Δ, and *rad3*Δ. Trendlines and R-squared values are provided in all graphs. The *rad3*Δ strain displayed a slight correlation, lacking in the wild-type and *cds1*Δ strains.

In all three strains studied, DNA re-replication varied regionally such that stretches of hundreds of kilobases were amplified during re-replication or were relatively under-replicated (Figure 3). Given that most of the genome is subject to re-replication, one possibility is that re-replication is primarily a repeat of S-phase. If so, then normal S-phase origins, unusually active individual S-phase origins, or clusters of S-phase origins might be determinants of which sequences are amplified during re-replication. To investigate this idea, we compared the re-replication profiles for all three chromosomes with various measures of S-phase origins (data not shown) including predicted origin distribution [34], distribution of 0.5-kb stretches of unusually high AT content, origin activity in HU-treated S-phase cells ([31,35-37]), early S-phase replication (KLM and JL, unpublished results), and localization of ORC and MCM proteins [37]. These comparisons led us to conclude that determinants of re-replication efficiency are distinct from normal S-phase replication. By way of example, Figure 5 shows comparisons of chromosome 1 re-replication with measures of S-phase replication and origin distribution. Figure 5A shows data for all of chromosome 1, while Figure 5B shows a magnified view of the 1-Mbp region highlighted in Fig. 5A. The large zones of re-replication common to wild-type, *cds1*Δ (not shown) and *rad3*Δ cells failed to show a clear one-to-one correspondence with S-phase replication in synchronous or HU-treated cells.

The more detailed view in Figure 5B shows that the peak of regional amplification is displaced about 50 kb toward the telomere from the local peaks of highest S-phase replication. The re-replication peak is aligned closely with the region AT1041+, which appears to have medium function as an S-phase origin. AT1041+ is flanked for long distances on both sides by regions that, according to microarray analyses, did not replicate significantly in cells entering S phase in the presence of HU [35-37,31].

If re-replication amplification peaks result from use of multiple origins in a region, then the center of amplification in the population need not be located at an origin but could lie between origins. It has been calculated that 80% of re-replication peaks lie within 10 kb of a pro-ARS in budding yeast, and re-replication peaks can in some cases be assigned to a single origin in this organism [12]. Correlation between re-replication peaks and S-phase origins is less clear in fission yeast, though likely origins can be identified in some cases such as AT1041+ described above. Figure 5 shows origin strength and distribution determined for S-phase replication in HU. To look at the effects of groups or clusters of origins, the numbers and activities of origins were evaluated in a sliding window of 100 kb. Even with this approach, there were differences between S-phase origins and re-replication, suggesting that the determinants of re-replication differ in some respects from the determinants of origin function during normal S-phase replication.

Finally, the sequences replicated during HU-arrested S phase and the sequences amplified during re-replication in wild-type and *cds1*Δ cells (Figure 5C) are poorly correlated (R^2 ^= 0.0128 and 0.023, respectively). As noted above, there are many smaller peaks of amplification in *rad3*Δ cells which aligned with clusters of active origins in the replication profile (Figure 5A, B). Consistent with this, there is a small degree of correlation between the sequences replicated during HU-arrested S phase and the sequences amplified after re-replication was induced in *rad3*Δ cells (Figure 5C; R^2 ^= 0.104). This correlation could be due to characteristics of re-replication in the absence of Rad3 or it could be an indirect result of ongoing division, possibly followed by "normal" S phases, in the *rad3*Δ mutant. Regardless of the explanation, our ability to detect such correlation in the *rad3*Δ mutant emphasizes the absence of similar correlation in wild-type and *cds1*Δ cells.

## Discussion

By using microarrays to measure copy number, we have found that over-expression in fission yeast of the Cdc18* replication initiation protein that has mutations in Cdk1 phosphorylation sites drove re-replication of broad regions throughout the genome, rather than driving abundant re-replication of just a few sequences. Re-replication was independent of the replication checkpoint mediated by Cds1. Re-replication also seems to be largely independent of the Rad3 checkpoint. However, our results obtained with *rad3*Δ cells are preliminary, due to the fact that these cells continue to divide even after re-replication is induced. In all three cell types (wild-type, *cds1*Δ and *rad3*Δ), we found amplified (relatively highly re-replicated) regions spanning hundreds of kilobases. These were distinct from the broad regions containing strong origins observed in normal S phase. Greatest amplification occurred near the fission yeast telomeres and closely matched the pattern of telomere-associated amplification observed during budding yeast re-replication [12,14].

### Re-replication and the cell cycle

In principle, re-replication can take place either within S phase (on segments of DNA that have already been replicated) or in G2 phase. In recently published genome-wide studies of re-replication in budding yeast, Green *et al*. [12] and Tanny *et al*. [14] employed cells arrested in G2/M with nocodazole to ensure absence of contributions from cells still in S phase. Both groups found that origin selection during re-replication induced in G2/M is markedly different from origin selection during S phase. Tanny *et al*. [14] showed that part of this difference could be attributed to the fact that, during re-replication, pre-RCs were re-loaded onto G2 chromatin at only a subset of the origins where they would normally be loaded during G1 in preparation for a normal S phase. In addition, Tanny *et al*. [14] concluded that many origins that did re-load pre-RCs were not subsequently selected to fire during re-replication. The nature of this additional restraint is not known. Both groups found that there was little or no correlation between S-phase origin efficiency or the time in S phase when an origin is normally functional and the likelihood of that origin being active for re-replication [12,14].

How can one explain the profound difference between re-replication induced during G2/M phase and normal replication during S phase replication? It is possible that G2- or M-phase-specific chromatin modifications or condensation might contribute to the re-replication program observed in nocodazole-arrested cells. To explore this possibility, Green *et al*. [12] also studied re-replication initiated during an S phase. They found that the S-phase re-replication patterns were partially similar to those generated by re-replication in G2/M. However, the peaks of S-phase re-replication corresponded somewhat better than those of G2/M re-replication to the positions and timings of origins used during normal S phase. A difference between re-replication during G2/M and re-replication initiated in S phase was that, during G2/M, telomeres were extensively re-replicated. In contrast, telomeres were under-amplified when re-replication was initiated during S phase [12].

For our study, we induced Cdc18* over-expression in logarithmically growing, unsynchronized cells. In this case, differences between re-replication and replication cannot be attributed to a mitotic-arrested state of the cells. Nevertheless, *S. pombe *showed major differences between re-replication and S-phase replication similar to those seen in nocodazole-arrested budding yeast. Furthermore, the amplification of telomeric regions during *S. pombe *re-replication resembles telomere-region amplification during re-replication in nocodazole-arrested budding yeast. The normally late replication of these regions might account for failure to detect their amplification when re-replication was induced during S phase in budding yeast. It is truly remarkable that parameters of re-replication should be so similar across the huge evolutionary distance between fission and budding yeasts and across the considerably different experimental designs.

Re-replication under the experimental conditions in this study appeared to require ~4 hours for a doubling of DNA content (compare the 17-hour and 21-hour time points in Fig. 1B), compared with a typical S-phase duration of ~40 minutes. Thus, within individual cells, there must either be large periods of time that are not permissive for re-replication, or re-replication must be inefficient relative to S-phase replication. The fact that *cds1*Δ and wild-type cells re-replicated with essentially identical kinetics (Fig. 1B) suggests that the explanation (whatever it may be) for the difference in DNA doubling times between normal S phase and our re-replication conditions is probably independent of the replication checkpoint.

We had not anticipated that *rad3*Δ cells would be able to divide during re-replication driven by overexpression of Cdc18*, because overexpression of wild-type Cdc18 can cause a Rad3-independent block to mitosis [38]. Our results are consistent with the model that the Cdc18* used in this study resembles a Cdc18 N-terminal deletion mutant, which cannot block mitosis independently of Rad3 [38]. Cdc18* lacks four of the five N-terminal Cdk1 phosphorylation sites, and analogous sites in budding yeast Cdc6 are required for Cdk1 inhibition [39]. It seems likely that Cdc18* is defective as a direct inhibitor of Cdk1 and so would depend on the Rad3 pathway to inhibit Cdk1 and prevent cells from entering mitosis. Because the *rad3*Δ cells were able to divide when overexpressing Cdc18*, while wild-type and *cds1*Δ cells were not, we conclude that Rad3 is important for detecting ongoing re-replication and for generating a checkpoint response capable of inhibiting cell division. Our data further indicate that Cds1 plays a small, but detectable, role in the checkpoint response to re-replication, because Cds1 was required to maintain the cell-cycle arrest in some cells. That most *cds1*Δ cells maintain the arrest is likely due to activation of the Chk1-dependent damage checkpoint pathway. Consistent with this idea, re-replicating cells lacking Chk1 also have partial defects maintaining cell cycle arrest (data not shown). These observations are consistent with reports that re-replication generates DNA damage and activates checkpoint responses in other eukaryotes [27,21,16,20,19,24,23,18,26].

### Regional re-replication

As Green *et al*. and Tanny *et al*. had found for nocodazole-arrested budding yeast [12,14], we found that amplification during re-replication was poorly correlated with S-phase replication in fission yeast. In contrast with the yeast results, a strong correlation was apparent between re-replication in a mammalian cell line and euchromatic sequences normally replicated during early S phase, based on using re-replicated DNA for FISH hybridization to metaphase chromosomes [27]. Thus the regional determinants of re-replication in eukaryotes still hold many mysteries.

Intriguingly, in fission yeast, budding yeast, and mammalian cells, re-replication was seen to affect large regions rather than applying evenly to the genome [12,14,27]. In fission yeast, we found that large regions of hundreds of kilobases were amplified, interspersed with large regions that re-replicated less than average. Broad regions are most simply explained as being the result of one or a few initiations near the region centers. Each amplified region would be replicated by forks moving outward from its center. If forks travel at 1 to 3 kb/minute, as calculated by Rivin and Fangman for budding yeast [40], or at 2.8 kb/minute as proposed by Heichinger *et al*. for *S. pombe *[36], then bi-directional forks starting at one origin could replicate 0.5–1.5 Mbp in four hours, the approximate time frame in which total DNA content doubles during re-replication in our study. This is consistent with the observed sizes of the amplified regions. The lack of correspondence between fission yeast re-replication peak centers and origins known to be very active in S phase could result from use of origins that are normally inefficient during S phase, or from stochastic use of several sites of initiation during re-replication – with the *caveat *that, for some of these origins, relative efficiency would be different between S phase and re-replication. The overall similarity between fission yeast and budding yeast re-replication suggests that re-replication-specific origin preference might be a general phenomenon rather than a special property of the model organism or the details of the experimental design.

### Minimal effect of the Cds1-dependent replication checkpoint during re-replication

Considering that re-replication is a form of replication, we initially expected that the replication checkpoint might be activated. We were therefore surprised to see that, for every characteristic that we measured, wild-type and *cds1*Δ cells proved to be similar or identical. Indeed, the only difference we could detect between the two cell types was a somewhat faster loss of mitotic arrest in *cds1*Δ cells compared to wild-type. The *cds1*Δ mutation had no detectable effect either on the degree of re-replication or on which regions were preferentially amplified (Figs. 3, 4).

Cds1 is important for replication fork stability and processivity when replication forks are hindered by DNA damage or starvation for dNTPs during normal S phase. Since re-replication presumably takes place under conditions that are not optimal for replication, we initially suspected that Cds1 might prove important for fork processivity or stability during re-replication. However, this expectation appears to have been incorrect (Figs. 3, 4). The fact that both the patterns and extents of re-replication were indistinguishable between *cds1*Δ and wild-type cells suggests that Cds1 played no unique role in replication fork stability or processivity during re-replication and suggests that the Cds1-dependent replication checkpoint may not be induced, or may be induced only slightly, during re-replication in fission yeast cells.

If re-replication is initiated during a 'G2-like' state of the cells, then absence of the replication checkpoint should be expected, since the Mrc1 protein – an essential mediator of the replication checkpoint – is cell-cycle regulated and is abundant only during S phase [41]. The idea that Cdc18*-driven re-replication is initiated during a G2-like state is also consistent with the finding that Cdc18*-driven re-replication in fission yeast more closely resembles re-replication in G2/M arrested budding yeast than re-replication initiated in S-phase in budding yeast.

### Damage at replication forks during re-replication

The absence of a robust replication checkpoint response to stabilize replication forks during re-replication may be partially or completely responsible for the re-replication-induced DNA damage that activates the damage checkpoint. Operation of the replication checkpoint during normal, unperturbed S phase is essential to prevent the accumulation of damaged DNA (reviewed in [42,43]). It seems likely, therefore, that – in the absence of fork stabilization by the replication checkpoint – damage would also accumulate during re-replication. An additional phenomenon that may contribute to DNA damage during re-replication is the likely collisions of faster forks with slower forks traveling in the same direction (forks chasing forks; [19]).

### Consequences of absence of the DNA damage checkpoint (in *rad3*Δ cells) during re-replication

The patterns of re-replication in *rad3*Δ cells were largely similar to those in wild-type and *cds1*Δ cells (Figs. 3, 5). However, some differences were evident (Figs. 3, 4, 5), including greater amplifications of sequences at centromeres, the mating-type locus, and the telomeres of chromosomes 1 and 2. These regions have in common the fact that they are heterochromatic. The common response of all the major heterochromatic regions (telomeres, centromeres and the mating-type locus) to the *rad3*Δ mutation after inducing DNA re-replication is somewhat surprising, since during normal S phase the telomeres, which replicate late and are checkpoint-regulated, behave very differently from the centromeres and mating type locus, which replicate early and are not checkpoint-regulated [32,33,31]. In future studies, it will be interesting to elucidate the mechanisms by which the Rad3 protein suppresses over-replication of these heterochromatic regions during Cdc18*-induced re-replication. Futures studies should also shed light on the mechanisms leading to different timing and checkpoint-response behaviors for telomeres (on the one hand) and centromeres and the mating-type locus (on the other hand) during normal replication.

The *rad3*Δ mutation increased not only the amount of amplification but also the sizes of the amplified regions at telomeres, from 50–75 kb in wild-type and *cds1*Δ cells to more than 100 kb in *rad3*Δ cells (Fig. 4). It is known that Rad3 but not Cds1 is important for heterochromatin formation in sub-telomeric regions as measured by gene silencing [44]. Therefore it is tempting to speculate that telomere-associated effects are due to a Rad3-specific function directly affecting the chromosomes rather than being an indirect effect of the loss of cell cycle control in *rad3*Δ cells. One caveat is that telomeric heterochromatin normally extends no further than about 20 kb into the sequenced regions at the ends of chromosomes 1 and 2 [45]. This is smaller than the region amplified in any of the three strains. It is possible that re-replication forks initiated within telomeric heterochromatin may extend into centromere-proximal non-heterochromatic regions.

### Telomeric amplification as a possible marker for re-replication

Telomeric regional amplification is the clearest similarity between re-replication in fission and budding yeasts. There are extensive similarities between the sub-telomeric regions of these two evolutionarily distant yeast species. The telomeres are localized near the nuclear envelope, they are transcriptionally silenced, they are late-replicating, replication is checkpoint responsive, and these regions are preferentially amplified during re-replication (this study, [12,14]). In both yeasts, the size of the specifically-amplified region is ~50 kb. In both yeasts, this amplification zone is followed by a region of under-replication, suggesting the possible presence of some type of barrier or transition zone, with possible biological significance. That the distinct pattern of re-replication adjacent to telomeres is conserved between the highly diverged fission and budding yeasts suggests that preferential amplification may be a basic feature of re-replication near telomeres and therefore may also occur in metazoans.

## Conclusion

Here we have presented the results of the first microarray analysis in fission yeast of re-replication induced by over-expression of the initiation protein, Cdc18*, a mutant Cdc18 lacking N-terminal Cdk phosphorylation sites. This is also the first microarray analysis in any organism to compare re-replication in wild-type cells with re-replication in checkpoint-mutant cells.

Wild-type and checkpoint-mutant cells re-replicated DNA throughout their genomes, but the extents of re-replication varied somewhat, with the result that broad regions of hundreds of kilobases were relatively over-re-replicated or under-re-replicated by approximately two-fold or less. The locations of these broad regions did not correspond to the locations of origins that fired efficiently during normal S phase.

There were no significant differences in the patterns or extents of re-replication between wild-type cells and cells deleted for Cds1, a downstream checkpoint kinase that is essential for the replication checkpoint. This observation suggested that the replication checkpoint might not function during re-replication under our experimental conditions (induction of re-replication in exponentially growing cells) – consistent with the possibility that re-replication under our experimental conditions took place primarily during G2 phase, when the replication checkpoint is known to be inoperative.

Similar to re-replicating cells lacking Cds1, re-replicating cells lacking Rad3 (which functions in all phases of the cell cycle and is essential for both the replication and damage checkpoints) accumulated broad regions that were relatively over-re-replicated or under-re-replicated and strongly resembled the patterns observed in wild-type and *cds1*Δ cells. In contrast to wild-type and *cds1*Δ cells, *rad3*Δ cells attempted mitotic division after induction of re-replication. As a result, the small differences that we noted between the *rad3*Δ strain and our other strains may reflect Rad3-specific functions directly affecting re-replication or may be the result of mitotic divisions and cell cycle progression.

In wild-type and *cds1*Δ cells, the telomeres of chromosomes 1 and 2 were highly amplified, and the amplification spread away from the telomere for ~50–75 kb. The DNA from ~100 to ~300 kb from the telomere tended to be under-amplified, suggesting the possible presence of a re-replication barrier in this region. This pattern (over-re-replication near telomeres followed by under-re-replication ~100 kb later) is very similar to the pattern seen in the distantly related budding yeast [12,14], suggesting that the same pattern may be conserved in other eukaryotic organisms.

Since several different genetic alterations, each of which leads to excess activity of the initiation proteins Cdt1 and/or Cdc6, can induce re-replication in mammalian cells [27,21,24,23,26], it is likely that induction of re-replication is one of the genome-destabilizing processes that can lead to cancer. This potential relationship to cancer adds to the inherent importance and interest of studies of re-replication in eukaryotic model organisms such as the yeasts.

## Methods

### Strain construction, cell culture, and over-expression of Cdc18* for re-replication experiments

All strains expressed cdc18* from a stable integration at the *ura4 *locus, designated *ura4-294::GST-cdc18-T4A (ura4*^+^) [4]. The "wild-type" strain is JLP515: cdc18* (*h*^+^*leu1-32 ura4-294::GST-cdc18-T4A (ura4*^+^) [4]. The *cds1*Δ strain is JLP1285: cdc18* cds1Δ (*h*^-^*leu1-32 ura4-294::GST-cdc18-T4A (ura4*^+^*) cds1::ura4*^+^), and the *rad3*Δ strain is JLP1288: cdc18* rad3Δ (*h*^+^*leu1-32 ura4-294::GST-cdc18-T4A (ura4*^+^*) rad3::ura4*^+^). These double-mutant strains were constructed by crossing JLP515 with strains from Anthony Carr: 1562 (*h*^-^*cds1:: ura4 leu1-32 ura4-D18*) and 6G (*h*^-^*rad3:: ura4 ade6-704 leu1-32 ura4-D18*). The *cds1:: ura4 *and *rad3:: ura4 *mutations were followed by HU sensitivity, and *ura4-294::GST-cdc18-T4A (ura4*^+^) was followed by sensitivity to media lacking thiamine.

For re-replication, strains were grown at 32°C in EMM supplemented with Leucine, Adenine, Uracil and Histidine (LAUH) + thiamine (B1) at 2.7 mg/l [4] to an OD_600 _of ~0.500, harvested and washed with water, and then inoculated at a calculated OD_600 _of ~0.006 into two flasks with EMM+LAUH, one with thiamine and one without. The cells were then grown at 32°C until harvest 17, 21, or 25 hours later. Harvested cells were stored at -80°C.

### DNA processing, labeling, and hybridizations

Frozen cell pellets consisting of 7.0 × 10^8 ^cells were washed with water and re-pelleted, resuspended in 200 μl breaking buffer (2% Triton X-100, 1% sodium dodecyl sulfate [SDS], 100 mM NaCl, 10 mM Tris-Cl, pH 8.0, and 1 mM EDTA, pH 8.0). An equal volume of phenol/chloroform/isoamyl alcohol (PCI) and glass beads was added to the cells, and the suspension was vigorously shaken with a mini-beadbeater (BioSpecs Products) for 1 minute, then put on ice for 2 minutes. The shaking and ice incubations were repeated twice more. One half-volume of 10 mM Tris, 1 mM EDTA, pH 8.0 (TE) was added to the samples and mixed briefly (Vortex). The samples were then centrifuged at 16,100 × g for 8 min at room temperature. The aqueous layers were then transferred to pre-spun phase-lock tubes (Eppendorf), equal volumes of PCI were added, and the samples were shaken briefly (Vortex). Samples were clarified by centrifugation. The supernatants were transferred to new tubes, an equal volume of chloroform/isoamyl alcohol was added, and samples were mixed briefly (Vortex). After clarification by centrifugation, the supernatants were transferred to new tubes, an equal volume of cold 100% ethanol, and NaCl to a final concentration of 50 mM were added. The samples were mixed briefly (Vortex), then incubated at -80°C for at least 30 minutes. The precipitated DNA was pelleted by centrifugation for 15 minutes at 16,100 × g (4°C). Supernatants were aspirated, and the DNA pellets were dried before resuspension in TE. RNA was removed by addition of RNAse A (Sigma) at a final concentration of 0.1 μg/μl and incubation at 37°C for at least 30 min. To remove contaminating proteins, Proteinase K (Roche) was added at a final concentration of 0.4 μg/ml, and samples were incubated at 55°C for 30 min. To selectively precipitate the DNA, ammonium acetate was added to 0.1 M followed by two volumes of cold 100% ethanol, mixing (Vortex), incubation at -80°C for 30 minutes, and centrifugation for 15 minutes at 16,100 × g (4°C). The supernatants were aspirated and the DNA pellets were washed with 70% ethanol and centrifuged again. The supernatants were removed, the pellets were dried, and the DNA was resuspended in TE.

Next the isolated DNA was labeled with aminoallyl-dUTP (aa-dUTP). The bead-beating with glass beads (previous paragraph) was sufficient to shear the DNA into fragments of ~500 bp, an appropriate size for random-primed labeling. Reactions containing 4 μg isolated genomic DNA, 10 μg random hexamers (MWG), and Klenow buffer (60 mM Tris-Cl, pH 7.0, 6.0 mM MgCl_2_, and 12 mM β-mercaptoethanol [Sigma]) were incubated at 100°C for 10 minutes, then quick-cooled in ice-water for 5 minutes. Then dNTPs were added (0.36 mM dATP, dGTP, dCTP (Invitrogen); 0.12 mM dTTP (Invitrogen), 0.24 mM aa-dUTP (Ambion) and 25 units of Klenow Fragment (3'→5' exo-; New England Biolabs) were added. The final reactions were mixed briefly and incubated at 37°C overnight.

Labeled DNA was recovered using the Qiaquick PCR purification kit (Qiagen), except that Qiagen wash and elution buffers were substituted, respectively, with phosphate wash buffer (5 mM KPO_4_, pH 8.5, 80% ethanol) and elution buffer (4 mM KPO_4_, pH 8.5). After the labeled DNA was purified, it was pelleted and dried in a SpeedVac (Savant). Then the labeled DNA was coupled to either Cy3 or Cy5 (Amersham) by resuspension of the aa-dUTP-labeled DNA in 4.5 μl of 0.1 M Na_2_CO_3_, pH 9.0, with an equal volume of NHS-ester Cy-dye, followed by an hour incubation at room temperature. The control DNA, cdc18* at +0 hrs (when cdc18* expression is repressed), was coupled to Cy5, and the experimental DNA was coupled to Cy3. Uncoupled dye was removed using the Qiaquick PCR purification kit (Qiagen), following the manufacturer's instructions.

For hybridizations, experimental DNA with 80 pmol Cy3 plus control DNA with 80 pmol Cy5 was resuspended in a hybridization solution consisting of 25% formamide, 5 × SSC, 0.1% SDS, and 100 μg/ml of sonicated salmon-sperm DNA. Hybridizations were performed under lifter cover slips (Erie Scientific) at 50°C in a humidified chamber for 16–20 hrs. The microarrays used in these experiments were created by the Leatherwood/Futcher microarray facility at Stony Brook University. Each microarray consists of 5,407 spots of 0.1- to 1.2-kb PCR products printed onto glass slides coated with aminopropylsilane (Erie Scientific). Hybridized arrays were washed by gently shaking in the following solutions for the times and temperatures indicated: two quick washes with 2 × SSC/0.1% SDS at 50°C, two 10-min washes with 2 × SSC/0.1% SDS at 50°C, two 10-min washes with 0.1 × SSC/0.1% SDS at 50°C, and four quick washes with 0.1 × SSC at room temperature. Arrays were dried by centrifugation and scanned using an Axon 4000B scanner, controlled by GenePix Pro 6.0 software, with a pixel size of 10 microns. Photomultiplier tube gains were subjectively adjusted during pre-scan to maximize effective dynamic range and to limit image saturation.

### Microarray data extraction and analysis

Data from microarray scans was extracted as previously described [46]. Background was subtracted from each signal. The experimental to control ratios (Cy3 to Cy5) were then normalized to a value of 1.0 for the genome average. Then the log_2 _of each experimental to control ratio was calculated. For each point, the results from multiple (up to four) independent hybridizations were averaged. If a single probe corresponded to multiple locations, the probe value was plotted at each of those locations. If multiple probes coded for the same location, the average value of all probes coding for that location was calculated and plotted for the single location. The averaged experimental to control ratios were converted from log space back into linear space. Probes which lacked data from multiple hybridizations or which had an average deviation greater than 0.2 were eliminated to reduce noise. The final values have been deposited in the ArrayExpress public repository under accession number E-MEXP-1128 [47]. We employed the version of the fission yeast genome that was available from the Sanger Centre in May, 2006 [48]. This version had stretches of 1000 N's inserted into the chromosomal sequences to fill in each of the five gaps between contigs that were present at that time. Graphs were prepared using IgorPro 5 software (WaveMetrics).

In Figure 3, re-replication profiles were constructed using the medians of averaged wild-type 21- and 25-hour time points across the 5 neighboring probes in the genome.

In Figure 5, replication profiles from synchronized wild-type cells released into S phase in the presence of 15 mM HU for 4 hours were graphed using data from Mickle *et al*. [31]. Normal S-phase replication profiles from wild-type cells were created using microarray data from *cdc25-22 *block and release experiments (AO, KLM and JL, unpublished). Probe values from hybridizations of cells 75 to 95 minutes post release from a G2 arrest were averaged together and used to create a normal S-phase replication profile. Origin efficiencies cited by Mickle *et al*. [31] were converted into numeric scores as follows; strong origin were assigned a value of 5, medium origins were assigned a value of 4, weak origins were assigned a value of 3, very weak origins were assigned a value of 2, and origins which were below the limits of detection were assigned a value of 1, and graphed. To calculate the density of active S-phase origins, the cumulative sums of origin efficiency scores in 100-kb windows were calculated every 10 kb across chromosome 1 and at each origin position in chromosome 1. For comparison of relative copy numbers during re-replication and replication under HU stress, Microsoft Excel was used to graph the data and to calculate R-squared values. For wild-type, the amount of replication under HU stress was compared to the amount of re-replication by plotting the average relative copy numbers under HU stress for 4 hours [31] against the average re-replicated to control DNA ratios for strains induced for 25 hours of cdc18* expression. For *cds1*Δ, the amount of replication under HU stress was compared to the amount of re-replication by plotting the average relative copy numbers under HU stress for 4 hours in a *cds1*Δ strain [31] against the average re-replicated to control DNA ratios for the *cds1*Δ strain induced for 25 hours of cdc18* expression. For *rad3*Δ, the amount of replication under HU stress was compared to the amount of re-replication by plotting the average relative copy numbers under HU stress for 4 hours in a *rad3*Δ strain [31] against the average re-replicated to control DNA ratios for the *rad3*Δ strain induced for 25 hours of cdc18* expression.

### Flow cytometry

~3 × 10^7 ^cells were harvested by centrifugation, and washed in 5 ml ice-cold water, resuspended in 10 ml ice-cold 70% ethanol and stored at 4°C until needed. 1.8 ml of fixed cells were resuspended in 5 ml 0.1 M HCl containing 2 mg/ml Pepsin (Sigma) and incubated for one hour at room temperature to reduce polar staining. Then cells were washed once in 5 ml 50 mM sodium citrate, pH 7.0, resuspended in 1 ml 50 mM sodium citrate, pH 7.0, supplemented with 500 μg/ml of RNaseA (Sigma), and incubated for two hours at 37°C. 0.5 ml of cells were next stained in 50 mM sodium citrate, pH 7.0, supplemented with 1 μM Sytox Green (Molecular Probes), sonicated for approximately 5 seconds, and immediately analyzed on a FACScan (Becton Dickinson).

### DAPI and Calcafluor staining

Cells were fixed with 70% ethanol at 0, 17, 21, and 25 hours after removal of thiamine, then were rehydrated with water and heat-fixed to slides. DAPI and Calcafluor (Sigma) were used to stain the nuclei and septa of cells, respectively. Cells were observed using a Zeiss phase-contrast, epifluorescence microscope under blue-filtered UV-light illumination. For each time point, 200–300 cells were classified based on the presence or absence of a septum and whether the cell had one or two nuclei.

## Authors' contributions

KLM helped plan the experiments, carried out most of the microarray experiments and analyses, and contributed to writing the manuscript. AO made the arrays, helped plan and establish microarray-based copy-number detection for this study, and helped plan some of the experiments. JAH helped plan the experiments and contributed to writing the manuscript. JL helped plan the experiments and contributed to the analyses and to writing the manuscript. All authors read and approved the manuscript.

## Supplementary Material

Additional file 1 **Microarray measurements of re-replicated to control DNA throughout chromosome 1**. The column "Probe_Center" shows the positions, on the fission yeast nucleotide sequence of May, 2006, of the centers of all our PCR probes. The next column, "Gene_Name", shows the names of the probes. In most cases, these are the names of the ORFs containing the probes. Most probes were located in the 3' ends of ORFs. The columns headed "AVG_X_Yhr" show the normalized averaged re-replicated-to-control DNA ratios for the indicated probe in strain X at Y hours after removal of thiamine. N/A designates a lack of an average re-replicated-to-control DNA ratio for a probe due to fewer than two good hybridizations or an average deviation >0.2 for that particular probe. The numbers shown here are plotted in Figures 2 and 5 and in Additional Files 4 and 5 for chromosome 1. Click here for file

Additional file 2 **Microarray measurements of re-replicated to control DNA throughout chromosome 2**. Similar to Additional File 1, but for chromosome 2. Click here for file

Additional file 3 **Microarray measurements of re-replicated to control DNA throughout chromosome 3**. Similar to Additional File 1, but for chromosome 3. Click here for file

Additional file 4 **Genome-wide microarray analysis of DNA re-replication over time in cells lacking Cds1**. Similar to Figure 2, but for *cds1*Δ cells. Click here for file

Additional file 5 **Genome-wide microarray analysis of DNA re-replication over time in cells lacking Rad3**. Similar to Figure 2, but for *rad3*Δ cells. Click here for file

Additional file 6 **Percents of binucleate cells in populations undergoing re-replication**. The nuclei and septa of wild-type, *cds1*Δ, and *rad3*Δ cells from 0 (control), 17, 21, and 25 hours post induction of Cdc18* were stained with DAPI and Calcafluor respectively. At each time point, 200 to 300 cells were observed, and the numbers of mono- and bi-nucleate cells were scored. The percent of binucleate cells in each population is depicted in this graph. The *rad3*Δ strain has a large number of binucleate cells even at the 17-hour time point, indicating a failure in cell-cycle arrest. Click here for file
