# Supplementary figures and images for: Checkpoint effects and telomere amplification during DNA re-replication in fission yeast
Source: BMC Mol Biol. 2007 Dec 21;8:119. doi: 10.1186/1471-2199-8-119 (PMC2265721; doi:10.1186/1471-2199-8-119)

A

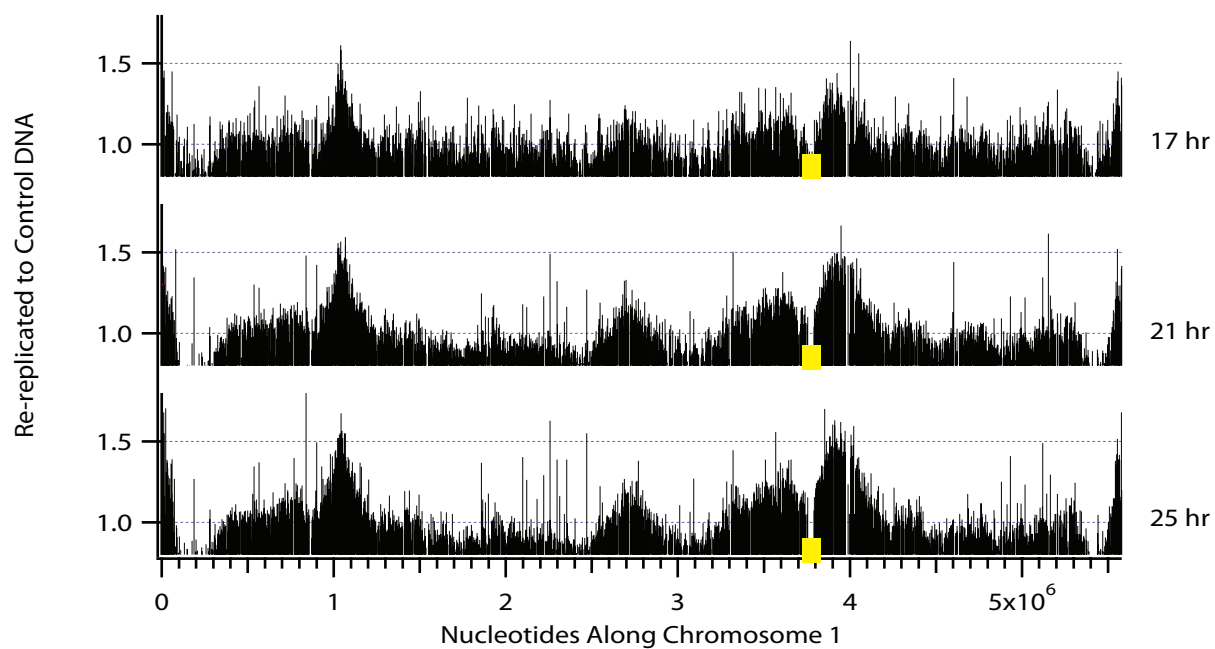

B

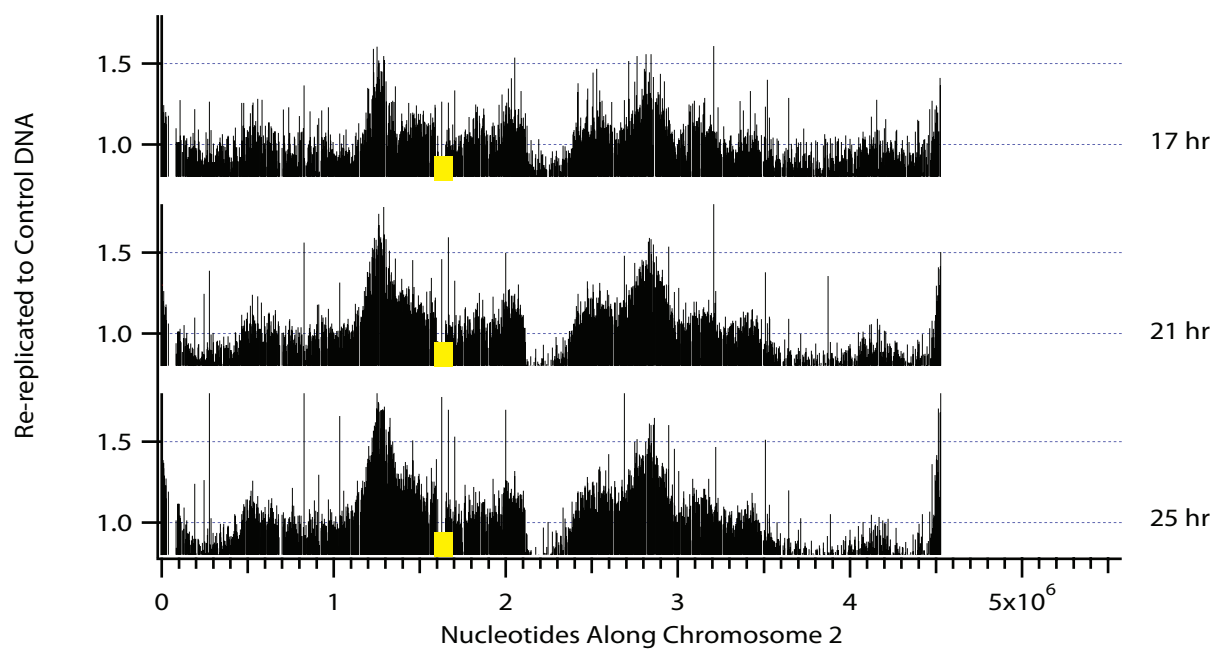

C

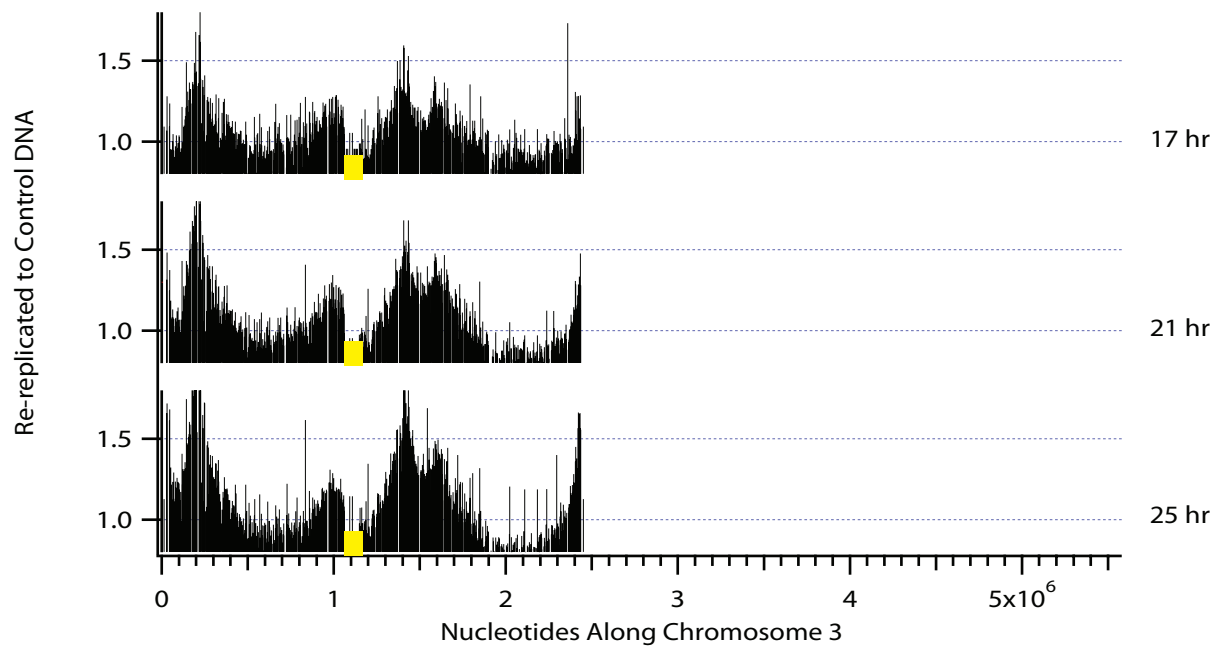

Supplement: Additional file 4 — Genome-wide microarray analysis of DNA re-replication over time in cells lacking Cds1. Similar to Figure 2, but for cds1Δ cells. [file 1471-2199-8-119-S4.PDF]

A

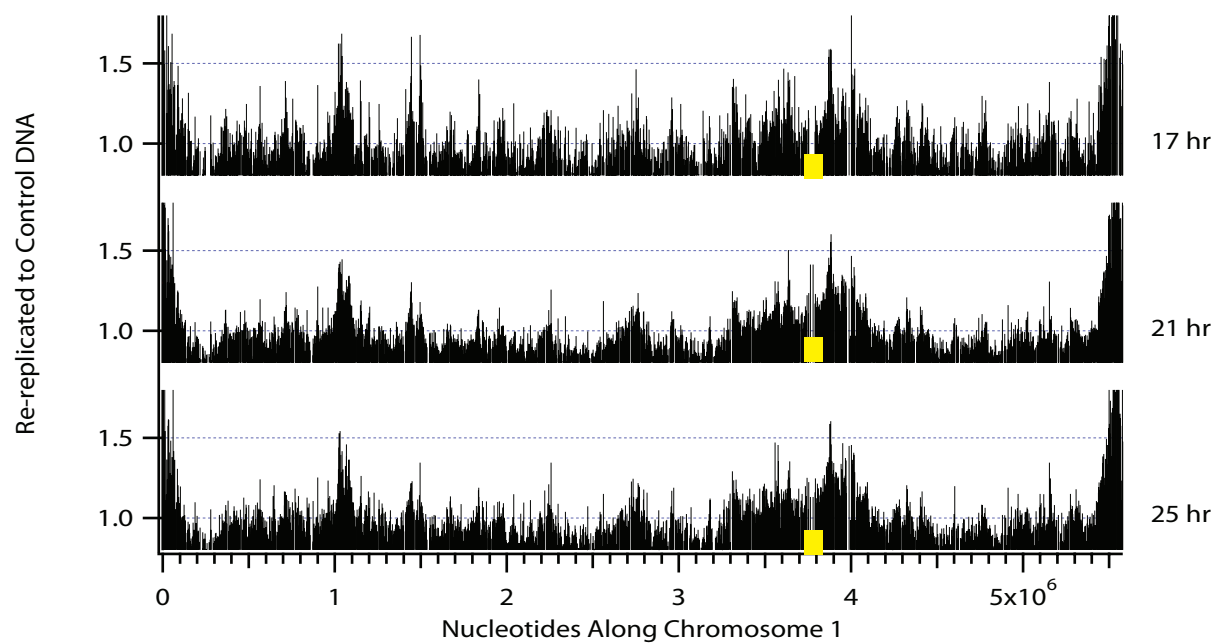

B

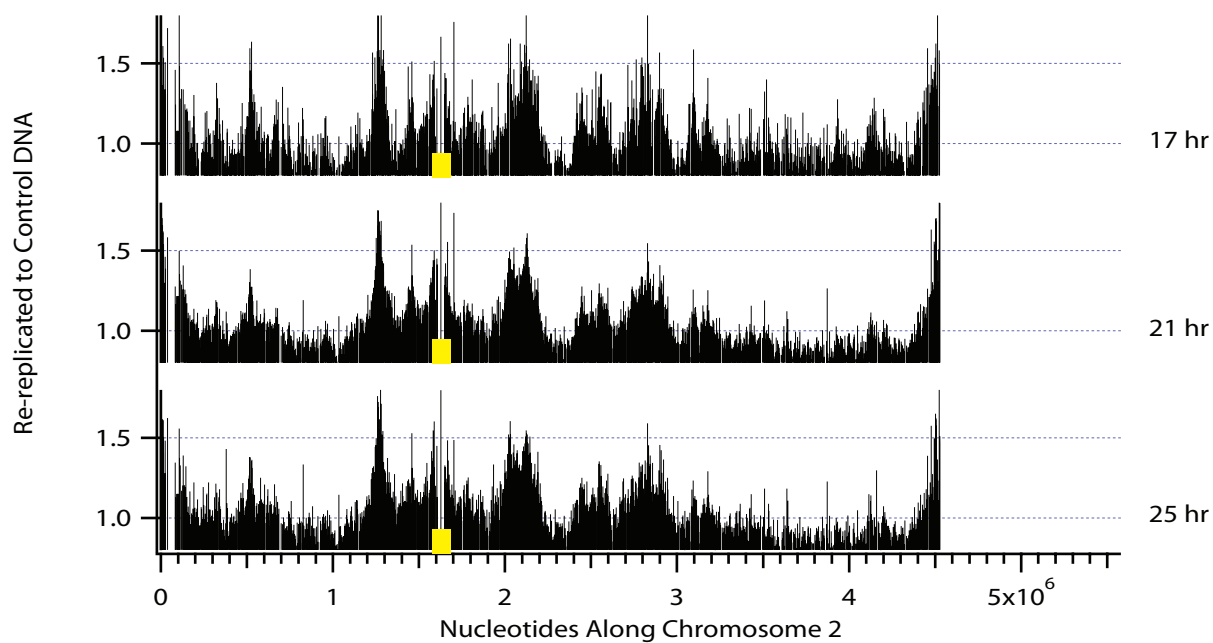

C

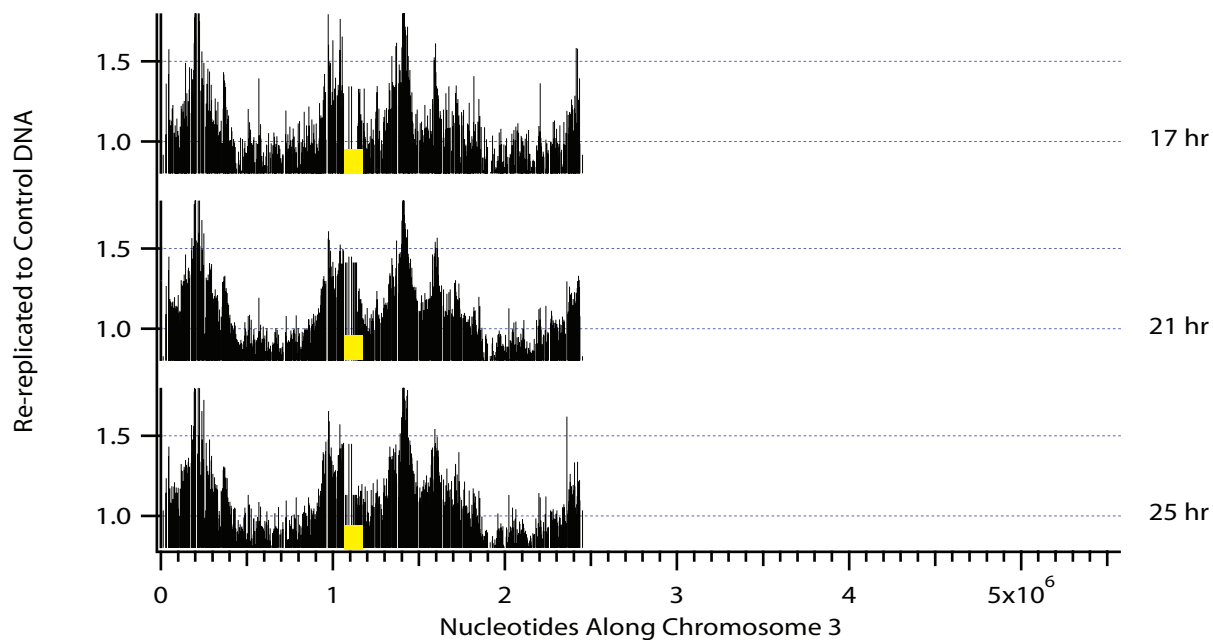

Supplement: Additional file 5 — Genome-wide microarray analysis of DNA re-replication over time in cells lacking Rad3. Similar to Figure 2, but for rad3Δ cells. [file 1471-2199-8-119-S5.PDF]

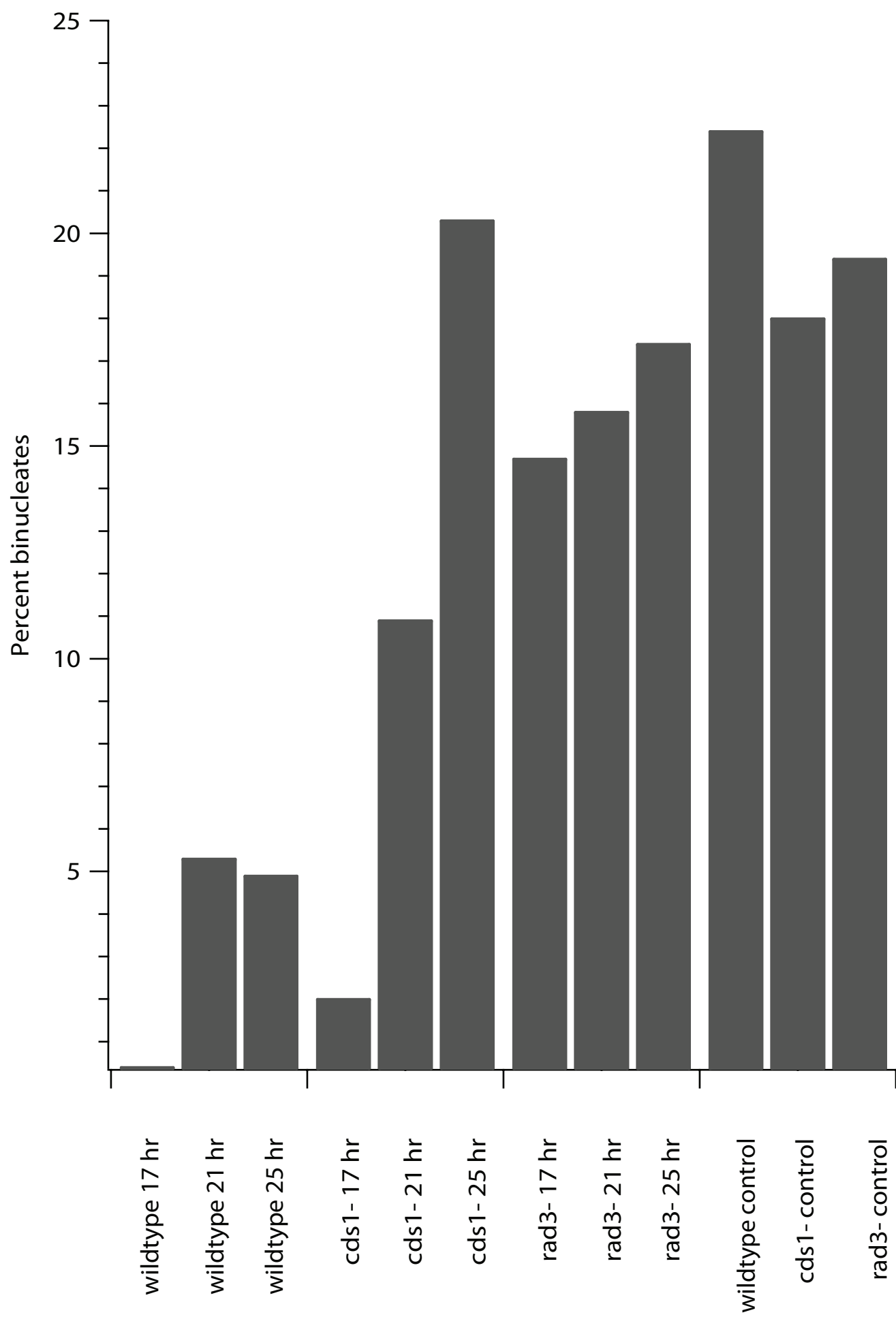

Supplement: Additional file 6 — Percents of binucleate cells in populations undergoing re-replication. The nuclei and septa of wild-type, cds1Δ, and rad3Δ cells from 0 (control), 17, 21, and 25 hours post induction of Cdc18* were stained with DAPI and Calcafluor respectively. At each time point, 200 to 300 cells were observed, and the numbers of mono- and bi-nucleate cells were scored. The percent of binucleate cells in each population is depicted in this graph. The rad3Δ strain has a large number of binucleate cells even at the 17-hour time point, indicating a failure in cell-cycle arrest. [file 1471-2199-8-119-S6.PDF]
